# Protein Buffering in Model Systems and in Whole Human Saliva

**DOI:** 10.1371/journal.pone.0000263

**Published:** 2007-02-28

**Authors:** Andreas Lamanda, Zeinab Cheaib, Melek Dilek Turgut, Adrian Lussi

**Affiliations:** 1 Department of Preventive, Restorative and Pediatric Dentistry, University of Bern, Bern, Switzerland; 2 Department of Pediatric Dentistry, Faculty of Dentistry, Hacettepe University, Ankara, Turkey; University of Munich, Germany

## Abstract

The aim of this study was to quantify the buffer attributes (value, power, range and optimum) of two model systems for whole human resting saliva, the purified proteins from whole human resting saliva and single proteins. Two model systems, the first containing amyloglucosidase and lysozyme, and the second containing amyloglucosidase and α-amylase, were shown to provide, in combination with hydrogencarbonate and *di*-hydrogenphosphate, almost identical buffer attributes as whole human resting saliva. It was further demonstrated that changes in the protein concentration as small as 0.1% may change the buffer value of a buffer solution up to 15 times. Additionally, it was shown that there was a protein concentration change in the same range (0.16%) between saliva samples collected at the time periods of 13:00 and others collected at 9:00 am and 17:00. The mode of the protein expression changed between these samples corresponded to the change in basic buffer power and the change of the buffer value at pH 6.7. Finally, SDS Page and Ruthenium II tris (bathophenantroline disulfonate) staining unveiled a constant protein expression in all samples except for one 50 kDa protein band. As the change in the expression pattern of that 50 kDa protein band corresponded to the change in basic buffer power and the buffer value at pH 6.7, it was reasonable to conclude that this 50 kDa protein band may contain the protein(s) belonging to the protein buffer system of human saliva.

## Introduction

When food enters our alimentary canal through the mouth, it first comes into contact with saliva, mainly excreted by the three major salivary glands. Acid containing beverages and foods as well as acids originating from the stomach are menaces to the teeth as these agents contribute to the erosion of tooth surfaces [1], [2]. Enamel and dentin are composed primarily of a carbonate substituted calcium deficient hydroxyapatite. When hydroxyapatite is in contact with water (saliva), hydroxyl ions (OH^−^) can remove from the tooth surface during an erosive challenge like drinking an apple juice [3], vomiting [4] or gastro-oesophageal reflux [5]. If this process is repeated frequently, a loss of tooth substance, also known as erosion, may be the consequence [6], [7]. Dissolution ends and remineralization of the dental hard tissues occurs when the pH in close proximity to the tooth begins to rise [2]. This rise in pH is caused by saliva that permanently covers the structures forming the oral cavity. The salivary components responsible for the increase in pH are the three buffer systems, carbonate, phosphate and protein buffer system [8], [9]. The carbonate and phosphate systems have been well characterized [8], [10], [11], [12], [13]. With the exception of the knowledge regarding that the total protein concentration varies from 0.15 to 0.65% [14] and that 940 different protein species are present in saliva [15], [16], [17], [18], [19], [20], [21], [22], the information about the protein buffer system is scarce [23], [24], [25], [26], [27].

Over the last 40 years, the prevalence of dental erosion increased continuously [28], [29]. As the buffer characteristics of saliva can influence the erosion process [3], the aim of the present study was to quantify the buffer characteristics of a model system for salivary protein buffering and proteins prepared from whole saliva.

## Materials and Methods

### Acid/base titrations

Ten milliliters (ml) of the analytes (saliva samples or solutions) were placed in a vessel in a water bath and stirred at 37°C. First, 5 ml of NaOH 0.01 mol/l were added in steps of 200 µl to enclose the buffer range of *di*-hydrogenphosphate (pH 6.1–8.1) and then 25 ml of HCl 0.01 mol/l were added in steps of 200 µl. The pH was measured with a micro glass pH electrode 3 mm in diameter (DG 101-SC, Mettler Toledo, Schwerzenbach, Switzerland) and recorded after each addition step. Data points were fitted with Sigmaplot V9.0. Buffer values β, in [mol/(l×pH) [30]], were calculated as β = −ΔC/ΔpH [31] where ΔC is the amount of the titrator used (acid/base) and ΔpH is the change in pH caused by the addition of the titrator. The buffer value was used to quantify the buffer capacity. The buffer optimum was determined at the pH with highest buffer value within the buffer range. The buffer range, in pH units, was used to describe the pH interval where the buffering reaction of one or a mixture of compounds took place. The buffer power B, in µmol (H^+^) and µmol (OH^−^), was used to quantify the amount of acid and base that can be buffered by a substance or a mixture of substances. The first derivative of the normalized titration curve (ΔC/ΔpH) was plotted against the pH and B was determined at the point with maximum slope. The experimentally measured values were compared to those that were calculated. The buffer power B [mol/l] was calculated according to the formula B = c^2^/2c, where c is the concentration, in mol/l, of the buffer component(s) [31]. Purified human salivary protein was analyzed by an automated titration system (Mettler-Toledo DL53, and the Software Lab X pro V 2.10.000) with the same titration parameters except that only 50 µl of acid were added per step.

### Control group (human saliva samples)

The saliva was collected using a widely accepted procedure [32] under resting conditions, between 9:00 am and 10:00 am, from unmedicated volunteers who refrained from eating, drinking, smoking and performing oral hygiene measures for 2 hours (hr) before collection. Prior to saliva collection, the procedures were explained to the patients and an informed consent was taken from each of them. After collection, the buffer capacity of the saliva samples was determined using the CRT®buffer test (Ivoclar Vivadent, Schaan, Lichtenstein) as follows: the entire reaction pad was wetted with saliva. The saliva excess was dropped off from the test strip. After 5 minutes (min) of reaction time, the final color of the reaction pad was compared to the color of the standard color code chart. The samples were subjected to titration immediately after collection to prevent discrepancies caused by protease activity and the formation of ammonium by urease. For protein precipitation saliva samples were collected following the same procedure as described above at 9:00 am, 13:00 and 17:00.

### Averaging saliva titration curves

Unstimulated saliva samples of 5 male subjects aged 35 to 45 with buffer capacities ranging from low to high according the CRT® buffer test were subjected to acid base titration as described above. The pH measurements were recorded with constant increments due to the monotone nature of the titration. As all pH measurement points in the saliva titration curves corresponded to each other, they were averaged and the standard deviation was calculated.

### Search for model proteins

First, the availability (>1 g) of high-pure water soluble proteins at reasonable costs (<100 €/g) was checked. In this regard, about 100 proteins were selected. Then, the isoelectric point (theoretical best buffering point) of the selected proteins was calculated with the ProtParam analysis tool [33] at www.expasy.org. The proteins with buffer optima beyond the buffer range of hydrogencarbonate and *di*-hydrogenphosphate (pI within the range pH 3 to 5 or pH 8 to 10) were selected (group A).

Secondly, a list containing all known human salivary proteins was created from the literature. Their isoelectric points were calculated with the ProtParam analysis tool [33]. The proteins with buffer optima beyond the buffer range of hydrogencarbonate and *di*-hydrogenphosphate (pI within the range pH 3 to 5 or pH 8 to 10) were selected (group B). The amino acid sequences of the proteins in group A were aligned against the amino acid sequences of the proteins in group B with BLAST [34] or LALIGN [35]. A sequence in group A was selected if more than 30% of its amino acid sequence was identical to the amino acid sequence of a protein in group B.

### Solutions

#### Inorganic buffer compounds

Water: 10 ml deionized water was used. *Di*-hydrogenphosphate solution: 0.68 g (5 mM) KH_2_PO_4_ (Merck, Dietikon, Switzerland, for analysis, M = 136.09 g/mol, pK_a_ = 7.1) was dissolved in 1000 ml deionized water. Hydrogencarbonate solution: 0.84 g (10 mM) NaHCO_3_ (Merck, for analysis, M = 84.01 g/mol, pK_a_ = 6.1) was dissolved in 1000 ml deionized water. Hydrogencarbonate and *di*-hydrogenphosphate solution: 0.68 g (5 mM) KH_2_PO_4_ and 0.84 g (10 mM) NaHCO_3_ were dissolved in 1000 ml deionized water.

#### Organic buffer compounds

Amyloglucosidase solutions: 10 µM (0.1%), 20 µM (0.2%), 50 µM (0.5%) amyloglucosidase from Aspergillus *niger* (Fluka BioChemika, Buchs, Switzerland Swissprot P69328, 640 amino acids, M*r* = 98 kDa, p*I* = 4.35) was used as a model for human α-amylase (Swissprot P04745, 511 amino acids, M*r* = 57.8 kDa, p*I* = 6.4). 0.01, 0.02 or 0.05 g amyloglucosidase were dissolved in 10 ml deionized water. Lysozyme solution: 340 µM (0.5%) lysozyme from hen egg white (Fluka BioChemika, Swissprot P00698, 147 amino acids, M*r* = 14.6 kDa, p*I* = 9.4) was used as a model for human salivary lysozyme (Swissprot P61626, 148 amino acids, M*r* = 16.5 kDa, p*I* = 9.4). 0.05 g lysozyme was dissolved in 10 ml deionized water. α-amylase solutions: 20 µM (0.1%), (40 µM) (0.2%) and 100 µM (0.5%) α-amylase from hog pancreas (Fluka BioChemika 10080, Swissprot P00690, 511 amino acids, M*r* = 57 kDa, p*I* = 6.5) was used as a model for human salivary α-amylase. 0.01 g, 0.02 g and 0.05 g were dissolved in 10 ml deionized water. Amyloglucosidase and lysozyme solution: 10 mg (0.1% 10 µM) amyloglucosidase and 50 mg (0.5%, 340 µM) lysozyme were dissolved in 10 ml deionized water. α-amylase and amyloglucosidase solutions: 0.01 g (0.1% 10 µM) amyloglucosidase and 0.02 g (0.2%, 40 µM) α-amylase from hog pancreas were dissolved in 10 ml deionized water. α-amylase and amyloglucosidase solution: 0.01 g (0.1% 10 µM) amyloglucosidase and 0.02 g (0.2%, 40 µM) α-amylase from hog pancreas were dissolved in 10 ml deionized water.

#### Combined organic and inorganic buffer compounds

Amyloglucosidase, lysozyme hydrogencarbonate and *di*-hydrogenphosphate solution: 0.01 g (0.1%, 10 µM) amyloglucosidase and 0.05 g (0.5%, 340 µM) lysozyme were dissolved in 10 ml of a solution containing 0.68 g (5 mM) KH_2_PO_4_ and 0.84 g (10 mM) NaHCO_3_ per 1000 ml deionised water. α-amylase, amyloglucosidase hydrogencarbonate and *di*-hydrogenphosphate solution: (0.1% 10 µM) amyloglucosidase and 0.05 g (0.5%, 100 µM) α-amylase from hog pancreas were dissolved in 10 ml of a solution containing 0.68 g (5 mM) KH_2_PO_4_ and 0.84 g (10 mM) NaHCO_3_ per 1000 ml deionized water. α-amylase, amyloglucosidase hydrogencarbonate and *di*-hydrogenphosphate solution: (0.1% 10 µM) amyloglucosidase and 0.02 g (0.2%, 40 µM) α-amylase from hog pancreas were dissolved in 10 ml of a solution containing 0.68 g (5 mM) KH_2_PO_4_ and 0.84 g (10 mM) NaHCO_3_ per 1000 ml deionized water. Salivary protein solution: The fresh prepared salivary proteins from 10 ml of stimulated saliva were dissolved in 10 ml deionized water. After adjustment of the pH to 7, all solutions were stored in gas-proof closed vessels.

### Precipitation and dialysis of salivary proteins

Ammoniumsulphate was added to 10 ml of fresh collected resting saliva under constant stirring at 0°C. When 75% of ammoniumsulphate saturation was reached the mixture was stirred for additional 30 min. After centrifugation at 14000 rpm on a Hicen 21 centrifuge (Jepson Bolton, Watford, England) for 30 min at 4°C, the supernatant was removed and the obtained precipitate was dissolved in 5 ml deionised water. To remove all inorganic ions, the solution was dialyzed (Sigma dialysis sacks D6191-25EA, Sigma, Buchs, Switzerland) overnight at 4°C against deionized water. After dialysis, the volume of the dialyzed solution was adjusted to 10 ml.

### Electrophoresis

Electrophoretic separation (SDS Page) was performed on a Mini-PROTEAN® 3 cell (BioRad, Rheinach, Switzerland) using a 17.5% polyacrylamide gel as previously described [36].

### Fast RuBPS polyacrylamide gel staining

The polyacrylamide gels used for protein separation were visualized with Ruthenium II tris-bathophenantroline disulfonate (RuBPS). RuBPS was synthesized according to Rabilloud [37] and the staining was done as previously described [36] with modifications. In brief: the gel was placed in 50 ml of 40% Ethanol/10% acetic acid containing 1 µM RuBPS for 1 hr. After 20 min of destaining in 40% Ethanol/10% acetic acid, the gel was washed for 10 min in water and then scanned with an Amersham Storm 860 scanner (Amersham Bioscience, Freiburg, Germany). Images were processed with the advanced image data analyzer software (AIDA, v4.10, Raytest, Straubenhardt, Germany).

### Determination of the protein concentration

The protein concentration was determined with the colorimetric method according to Bradford [38].

## Results

### Search for human salivary α-amylase and lysozyme substitutes

15 proteins fitted to the selection criteria of group A and 346 proteins of group B. Three proteins were chosen to serve as model proteins: lysozyme from hen egg which has an isoelectric point of 9.4 and 57% sequence similarity to human salivary lysozyme, amyloglucosidase from Aspergillus *niger* which has an isoelectric point of 4.35 and 35% sequence similarity to human α-amylase and hog pancreatic α-amylase which has an isoelectric point of 6.5 and 86% sequence similarity to human α-amylase. Among the α-amylases of all species, hog pancreatic α-amylase has the closest affinity to human salivary α-amylase. Therefore only the high sequence similarity was taken in account.

### Inorganic buffer compounds

#### Water

**Figure 1:**
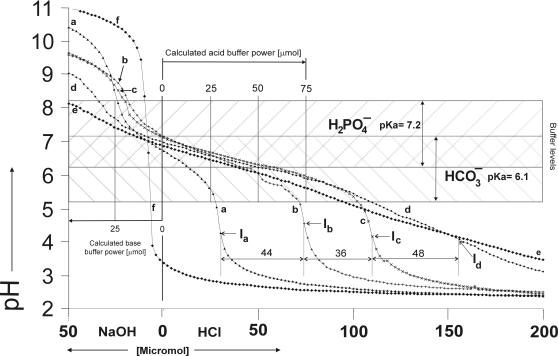
Titration curves with 150 pH measurements per curve of (a) 5 mM *di*-hydrogenphosphate, (b) 10 mM hydrogencarbonate, (c) 10 mM hydrogencarbonate plus 5 mM *di*-hydrogenphosphate, (d) 10 µM (0.1%) amyloglucosidase, 340 µM (0.5%) lysozyme, 10 mM hydrogencarbonate and 5 mM *di*-hydrogenphosphate (model system I), (e) 10 µM (0.1%) amyloglucosidase, 40 µM (0.2%) α-amylase, 10 mM hydrogencarbonate and 5 mM *di*-hydrogenphosphate (model system II) and (f) deionized water. The calculated buffer power is indicated in µmol per 10 ml of the analytes, in the internal scale.

Water (Fig. 1,f) was found to have no measurable buffer power or buffer value.

#### 5 mM *di*-hydrogenphosphate

A solution of 5 mM *di*-hydrogenphosphate (Fig. 1,a) was found to have buffer power of 30 µmol acid (hydrogen ions, H^+^) and 24 µmol base (hydroxyl ions, OH^−^). Optimal buffering was measured at pH 6.7 with 0.003 mol/(l×pH). The calculated buffer power was 25 µmol acid and base.

#### 10 mM hydrogencarbonate

A solution of 10 mM hydrogencarbonate (Fig. 1,b) was found to have a buffer power of 74 µmol (H^+^) and 8 µmol (OH^−^). Optimal buffering was measured at pH 6.2 with 0.005 mol/(l×pH). The calculated buffer power was 50 µmol acid and base.

#### 10 mM hydrogencarbonate plus 5 mM *di*-hydrogenphosphate

A solution of 10 mM hydrogencarbonate plus 5 mM *di*-hydrogenphosphate (Fig. 1,c), was found to have a buffer power of 110 µmol (H^+^), and 22 µσmol (OH^−^). The distances between inflections I_a_ and I_b_ as well as between I_b_ and I_c_ were larger than the calculated 25 µmol (H^+^). Optimal buffering was measured at pH 6.5 with 0.008 mol/(l×pH). The calculated buffer power was 75 µmol acid and base.

### Organic buffer compounds

#### 10 µM (0.1%), 20 µM (0.2%), 50 µM (0.5%) amyloglucosidase

**Figure 2:**
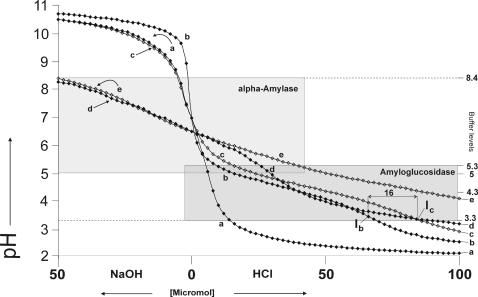
Titration curves with 86 pH measurements per curve of (a) 340 µM (0.5%) lysozyme in water, (b) 10 µM (0.1%) amyloglucosidase in water, (c) 10 µM (0.1%) amyloglucosidase plus 340 µM (0.5%) lysozyme in water, (d) 40 µM (0.2%) α-amylase and (e) 10 µM (0.1%) amyloglucosidase, 40 µM (0.2%) α-amylase.

**Figure 3:**
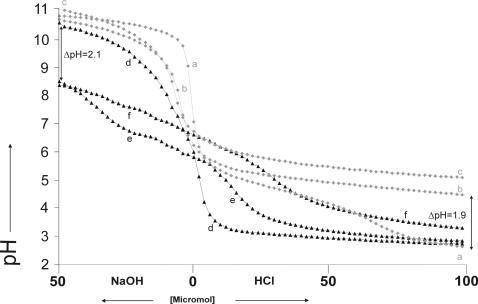
Titration curves with 80 pH measurements per curve of amyloglucosidase in concentrations (a) 10 µM (0.1%), (b) 20 µM (0.2%) and (c) 50 µM and α-amylase in concentrations (d) 20 µM (0.1%), (e) 40 µM (0.2%) and (f) 100 µM (0.5%).

A solution of 10 µM (0.1%) amyloglucosidase in water (Fig. 2,b, Fig 3,a) was found to have a buffer power of 68 µmol (H^+^) and 0 µmol (OH^−^). The buffer range spanned from pH 3.3 to 5.3 with optimal buffering at pH 4.35 and a buffer value of 0.004 mol/(l×pH). A solution of 20 µM (0.2%) amyloglucosidase in water (Fig. 3,b) was found to have a buffer power of 140 µmol (H^+^) and 0 µmol (OH^−^) and a buffer value of 0.02 mol/(l×pH) at pH 4.35, and a solution of 50 µM (0.5%) amyloglucosidase (Fig. 3,c) was found to have a buffer power (H^+^) that was out of the scale of this experiment (>200 µmol (H^+^)) and 0 µmol (OH^−^) with a buffer value of 0.02 mol/(l×pH) at pH 4.35. The increase in the amyloglucosidase concentration from 0.1% to 0.2% increased the buffer value 7 times at pH 4.5.The pH difference (ΔpH) between the 0.1% and 0.2% titration curves (Fig. 3 curves a and b) was 1.9 pH units after addition of 100 µmol acid and 0.2 pH units after addition of 50 µmol base.

#### 340 µM (0.5%) lysozyme

A solution of 340 µM (0.5%) lysozyme in water (Fig. 2,a) was found to have no measurable buffer attributes, although the protein had 32 titrable groups [39].

#### 20 µM (0.1%), 40 µM (0.2%), 100 µM (0.5%) α-amylase

A solution of 20 µM (0.1%) α-amylase in water (Fig 3,d) was found to have a buffer power of 54 µmol (H^+^) and 4 µmol (OH^−^). The buffer range spanned from pH 5 to 7 with optimal buffering at pH 6.3 and a buffer value of 0.0005 mol/(l×pH) and from 7.2 to 9 with optimal buffering at pH 8.4 and a buffer value of 0.0006 mol/(l×pH). A solution of 20 µM (0.2%) α-amylase in water (Fig. 2,d and 3,e) was found to have a buffer power of 66 µmol (H^+^) and 22 µmol (OH^−^) with a buffer value of 0.003 mol/(l×pH) at pH 6.3 and a buffer value of 0.01 mol/(l×pH). A solution of 50 µM (0.5%) α-amylase (Fig. 3,f) had a buffer of 80 µmol (H^+^) and 24 µmol (OH^−^), and a buffer value of 0.004 mol/(l×pH) at pH 6.3. In the 0.1, 0.2 and 0.5% α-amylase solution buffering was measurable from pH 5 to 8.4. The increase in the α-amylase concentration from 0.1% to 0.2% increased the buffer value 6 times at pH 6.3 and 15 times at pH 8.5. The pH difference (ΔpH) between the 0.1% and 0.2% α-amylase titration curves (Fig. 3, d and e) was 0.1 pH units after addition of 100 µmol acid and 2.1 pH units after addition of 50 µmol base.

#### 10 µM (0.1%) amyloglucosidase and 340 µM (0.5%) lysozyme

A solution of 10 µM (0.1%) amyloglucosidase and 340 µM (0.5%) lysozyme in water (Fig. 2,c) was found to have a buffer power of 84 µmol (H^+^) and 0 µmol (OH^−^). The buffer range spanned from pH 3.3 to 5.3 with an optimal buffering at pH 4.5 and a buffer value of 0.007 mol/(l×pH).

#### 10 µM (0.1%) amyloglucosidase and 40 µM (0.2%) α-amylase

A solution of 10 µM (0.1%) amyloglucosidase and 40 µM (0.2%) α-amylase (Fig. 2,e) was found to have an acidic buffer power of 114 (H^+^) and 8 µmol (OH^−^) with a buffer optima at pH 4.5 and a buffer value of 0.007 mol/(l×pH). Buffering was measurable between pH 3.5 to 5.5.

#### Buffering of purified salivary proteins

**Figure 4:**
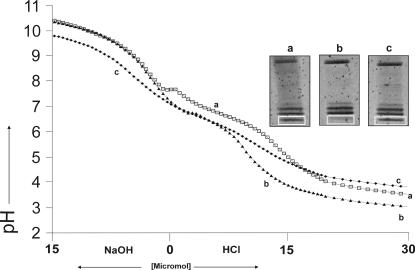
Titration curves with 80 pH measurements per curve of purified salivary protein from 10 ml saliva. Saliva samples were taken at (a) 9:00 am, (b) 13:00 and (c) 17:00. Next to the titration curves the corresponding electropherograms sections containing proteins from 50 to 110 kDa are shown. Proteins were visualized by modified ruthenium (ii) tris bathophenantroline staining.

10 ml of a solution containing the purified proteins from 10 ml whole stimulated human saliva collected at 09:00 am (Fig. 4,a) was found to have a buffer power of 11 µmol (H^+^, 100%) and 5 µmol of (OH^−^, 100%). 10 ml of a solution containing the purified proteins from 10 ml whole stimulated human saliva collected at 13:00 (Fig. 4,b), was found to have a buffer power of 9 µmol (H^+^, 82%) and 3 µmol of (OH^−^, 60%). 10 ml of a solution containing the purified proteins from 10 ml whole stimulated human saliva collected at 17:00 (Fig. 4,c), was found to have a buffer power of 8 µmol (H^+^, 73%) and 6 µmol of (OH^−^, 120%). The buffer range reached from pH 5 to 8 with a buffer optimum in all three samples at pH 6.7. The buffer value was 0.0008 mol/(l×pH, 100%) in the 09:00 sample, 0.0005 mol/(l×pH, 63%) in the 13:00 sample and 0.0008 mol/(l×pH, 100%) in the 17:00 sample. The protein concentration was 1.83 g/l in the 09:00 am and 17:00 sample and 1.76 in the 13:00 sample. The difference between the 9:00 am and 17:00 sample and the 13:00 sample was 0.16%.

### Combined inorganic and organic buffer compounds

Model system I, a solution of 10 µM (0.1%) amyloglucosidase and 340 µM (0.5%) lysozyme, 10 mM hydrogencarbonate and 5 mM *di*-hydrogenphosphate (Fig. 1 curve d, 5A curve b, 5B curve b) was found to have a buffer power of 158 µmol (H^+^) and 38 µmol of base. There were two discrete buffer optima within the buffer range between pH 3.4 to 7.5. The first was at pH 4.3 with a buffer value of 0.005 mol/(l×pH), whereas the second was at pH 6.5 with a buffer value of 0.01 mol/(l×pH).

Model system II, a solution of 10 µM (0.1%) amyloglucosidase and 40 µM (0.2%) α-amylase, 10 mM hydrogencarbonate and 5 mM *di*-hydrogenphosphate (Fig. 1 curve e, 5A curve c, 5B curve c) was found to have a buffer power of 132 µmol (H^+^) and 45 µmol (OH^−^). The solution had a buffer zone from pH 3.5 to 8 with buffer values starting from 0.004 mol/(l×pH) at pH 3.5 ascending to 0.008 mol/(l×pH) at pH 6.4 and descending to 0.003 mol/(l×pH) until pH 8.

**Figure 5:**
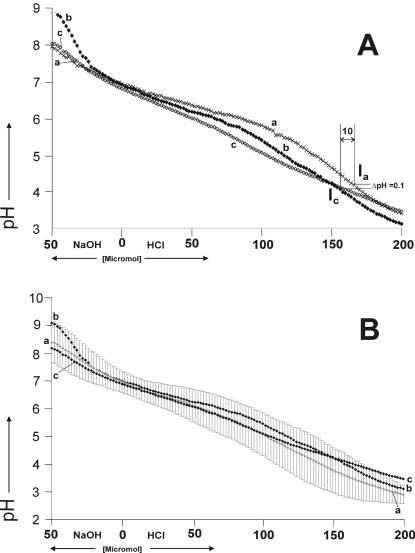
Panel A: Titration curves with 150 pH measurements per curve of (a) human saliva, (b) 10 µM (0.1%) amyloglucosidase, 340 µM (0.5%) lysozyme, 10 mM hydrogencarbonate and 5 mM *di*-hydrogenphosphate (model system I) and (c) 10 µM (0.1%) amyloglucosidase, 40 µM (0.2%) α-amylase, 10 mM hydrogencarbonate and 5 mM *di*-hydrogenphosphate (model system II). Panel B: Titration curve with 150 pH measurements per curve of (a) titration curve with 150 averaged pH measurements (5 per pH measurement point) of 5 male subjects with standard deviations indicated by grey bars. (b) 10 µM (0.1%) amyloglucosidase, 340 µM (0.5%) lysozyme, 10 mM hydrogencarbonate and 5 mM *di*-hydrogenphosphate, (c) 10 µM (0.1%) amyloglucosidase, 40 µM (0.2%) α-amylase, 10 mM hydrogencarbonate and 5 mM *di*-hydrogenphosphate (model system II).

Human resting whole saliva (Fig. 5A,a) was found to have a buffer power of 168 µmol (H^+^) and 42 µmol (OH^−^). Human resting whole saliva had a buffer zone from pH 3.4 to 8 with buffer values starting from 0.005 mol/(l×pH) at pH 3.4 ascending to 0.01 mol/(l×pH) at pH 6.5 and descending to 0.004 mol/(l×pH) until pH 8.

The average of the human resting whole saliva collected from 5 individuals (Fig. 5B,a) was found to have a buffer power of 154 µmol (H^+^) and 36 µmol (OH^−^). Average human resting whole saliva had a buffer zone from 3.5 to 8 with buffer values starting with 0.004 mol/(l×pH) at pH 4 ascending to 0.008 mol/(l×pH) at pH 6.5 and descending to 0.003 mol/(l×pH) until pH 8.

### Electrophoresis

The electropherograms of purified salivary proteins obtained from the 3 whole saliva samples collected at 9:00, 13:00 and 17:00 showed 32 protein bands of molecular weights from 14 kDa to 250 kDa (Fig. 4). 31 protein bands had unchanged band intensity whereas one 50 kDa protein band showed changed intensity. After subtraction of the background the numerically integrated band areas of the 50 kDa protein (Fig 4a,c) were 10876 LAU (linear arbitrary units, 100%) at 9:00 am to 4476 LAU (40%) at 13:00 and 9989 LAU (90%) at 17:00.

## Discussion

In the present study, the buffer attributes (value, power, range and optimum) of two model systems for human saliva, purified salivary proteins and single proteins were quantified by acid base titration. In the first step, the procedure was done by dissolving each of the model compounds in water separately. In the second step, the same compounds having the concentration as in the human saliva were mixed. In the third step, the procedure was done with human saliva and purified human salivary proteins. Then, the data obtained from the model systems, human saliva, purified salivary protein and single protein were compared. Amyloglucosidase from A. *niger*, lysozyme from hen egg and α-amylase from hog pancreas were used as model proteins because purified genuine or recombinant expressed salivary proteins were not available in the desired purity, quantity or at reasonable costs. α-amylase from hog pancreas has almost the same amino acid sequence as human salivary α-amylase and is its closest relative. Amyloglucosidase and lysozyme have the ideal physicochemical properties to demonstrate buffering beyond the buffer ranges of *di*-hydrogenphosphate and hydrogencarbonate. Amyloglucosidase and lysozyme have a high amino acid sequence similarity to their human counterparts whereas lysozyme from hen egg has almost the same molecular mass and the same isoelectric point as human salivary lysozyme. Moreover, this approach was feasible as the buffer function of a protein is dependent on its isoelectric point but independent from its catalytic properties or the species where it originates from. The total amyloglucosidase and lysozyme concentration in model system I (0.6%) as well as the total amyloglucosidase and α-amylase concentration in model system II (0.3%), did not exceed the total protein concentration found in human saliva [14].

346 human salivary proteins had their buffer optima beyond the buffer range of hydrogencarbonate and *di*-hydrogenphosphate (pH 5.1 to 8.1) what pointed out the plausibility that buffering beyond pH 5.1 to 8.1 could be based on proteins. Finally, buffering in saliva is likely to occur from proteins as in the rest of the human body where proteins are the most potent buffer substances [40].

In the present study, the experimentally determined buffer attributes of 5 mM *di*-hydrogenphosphate and 10 mM hydrogencarbonate were in agreement with the published data [8], [31] except for the observation that the carbonate system buffered 48% more acid than expected by calculation. The reason for this finding was attributed to the open system and is in agreement with the published data [30], [41].

Human whole saliva had a buffer zone spanning from pH 3.4 to 8 compassing the buffer ranges of hydrogencarbonate (pH 5.1 to 7.1) and *di*-hydrogenphosphate (pH 6.1 to 8.1). However, buffering in the range of pH 3.4 to 5 was not attributed to the buffering of hydrogencarbonate or *di*-hydrogenphosphate. It is known that at pH 4.3, hydrogencarbonate and *di*-hydrogenphosphate exhibit a maximum of 3% of their optimal buffer values [31]. The buffer values of the saliva samples measured in this study were in agreement with those published by Bardow [8] and even high concentrations of *di*-hydrogenphosphate and/or hydrogencarbonate can exhibit little buffer effect at pH 4.3 [31]. Therefore, it would be reasonable to conclude that we had the evidence that salivary buffering at pH 4.3 could be derived from the proteins.

The results of this study showed that the buffer value at pH 4.3 of model system I was 20 times higher than expected from 5 mM *di*-hydrogenphosphate and 10 mM hydrogencarbonate. However, at pH 4.3 model system I had exactly the same buffer value as human saliva and a buffer power that varied very little compared to the human saliva. The buffer value of model system II at pH 4.3 was 18 times higher than expected from 5 mM *di*-hydrogenphosphate and 10 mM hydrogencarbonate and almost identical to model system I and human saliva. This study showed that the purified salivary protein from 10 ml of whole saliva had the same buffer value at pH 5 as 5 mM *di*-hydrogenphosphate and 50% of the buffer value measured for 10 mM hydrogencarbonate. At pH 4.5, the buffer value measured for the salivary proteins was two times higher than for 5 mM *di*-hydrogenphosphate and 10 mM hydrogencarbonate. At pH 4, the buffer value measured for the salivary proteins was 6 times higher than for *di*-hydrogenphosphate and hydrogencarbonate where as at pH 3.5 the buffer value measured for the salivary protein was 6 times higher than for *di*-hydrogenphosphate and 4.5 times higher than for hydrogencarbonate.

For the combination of hydrogencarbonate and *di*-hydrogenphosphate with amyloglucosidase and lysozyme, 75% of the buffer value at pH 6.5 derived from hydrogencarbonate and *di*-hydrogenphosphate. The remaining 25% derived from amyloglucosidase and lysozyme. These results were unexpected as the fraction of the buffer value derived from proteins were responsible for only 3% of the buffer value at pH 6.5 [31]. Therefore, these findings were concluded as the evidence of the contribution of proteins to a larger fraction of the buffer value at pH 6.5 than hitherto assumed. These results, therefore, both support the hypothesis of Sellmann [42] regarding that proteins buffer at low pH values and the assumption of Freidin [25] who proposed protein buffer activity in a zone from pH 5.5 to 7.8.

The results of this study showed that a change in protein concentration (e.g. α-amylase) as small as 0.1% may change the buffer power up to two times and the buffer value up to 15 times. This change was within the same range as measured for the total protein content of the saliva samples taken at 9:00 am and 17:00 that had a 0.16% higher concentration than the samples taken at 13:00. As only one 50 kDa protein band of a total of 32 protein bands showed a lower intensity in the samples taken at 13:00 it was reasonable to conclude that this changed band caused the chances in basic buffer power and the buffer value at pH 6.7. Two time repetition of the experiments confirmed these results.

The 50 kDa protein band was subjected to protein identification which was performed by mass spectrometry and peptide mass fingerprinting (results not shown). Two proteins, α-amylase and serum albumin were identified. Although there are isoenzymes of α-amylase known with a masses around 50 kDa [43], the identification did not reach significance level. This was also the case for serum albumin. As SDS Page probable cannot provide high enough resolution to separate the different protein species that may be present in single protein band, further studies applying 2D electrophoresis will be necessary for unambiguous protein identification. In this study, the role of carbonic anhydrases and urease was neglected because both enzymes are found mainly in the enamel pellicle [9], [44] which was not included in the experiments.

The present study demonstrated that salivary buffering between pH 3.4 and 5 was not based on hydrogencarbonate and *di*-hydrogenphosphate but rather on proteins. Buffering between pH 5.1 and 8 was found to be based mostly on hydrogencarbonate and *di*-hydrogenphosphate but also seemed to be dependent on a larger fraction of proteins than thought before [8], [23]. There is some evidence that α-amylase could be one of the protein buffers in human saliva. In this context, it is worth mentioning the recently discovered human salivary α-amylase subproteom which consists of 67 amylase subspecies with isoelectric points ranging from pH 3.5 to 7.6 [43]. These α-amylase variants may provide like zwitterionic buffers [45], [46], a buffer system operational between pH 3.5 and 5 and auxiliary buffering through anionic and cationic sites present as non-interacting carboxylate and ammonium side chains between pH 5 and 8. However, further studies have to be undertaken to identify the protein buffer components in the human salivary proteome. The “Bufferomic” approach, as demonstrated in this article, is maybe only the first step in this direction.
